# Double Optic Neuritis

**Published:** 1896-03

**Authors:** J. M. Krim

**Affiliations:** Louisville, Ky.


### DOUBLE OPTIC NEURITIS.

REPORTED TO THE LOUISVILLE CLINICAL SOCIETY

BY J. M. KRIM, M. D., LOUISVILLE, KY.

I present this gentleman, Mr X, age forty-five years, for your examination; his case is interesting as well as unique, as far as my experience goes. I have had him under observation about four weeks. According to the history, I have been able to elicit, ten months ago, while sewing with a sewing machine, he was seized with intense pain in the abdomen. Three-quarters of an hour afterward, a physician saw him and administered remedies which afforded relief, but an hour later the patient became blind and remained so for two or three hours. He gradually regained vision, but it was somewhat blurred, and from that time on, at intervals, he has suffered with intense headache, starting, as he says, in the back part and coming forward to about the center of the cranium. He has been under treatment of various physicians in this city, and in Cincinnati, without any permanent relief; that is, he would be partially free from pain for two or three days; and sometimes, for a few hours, he would be entirely free from headache. For six weeks, he says, he had no pain; it then came on again, and he has not been free from it since. His eyesight was not completely restored, but he could see sufficiently well to follow his work.

About five weeks ago, he suddenly became totally blind and has remained so ever since. His headache is, to some extent, relieved, but it comes on occasionally, lasting for half an hour to an hour, then leaves him again. He has been under treatment with electricity in Cincinnati, galvano-cautery, blistering; he has been given iodide of potassium, etc. Several oculists of this city have examined him, and they all come to the same conclusion—that he has double optic neuritis. As far as I get the family history from the patient, both his parents died from old age. There is no evidence of specific trouble. He has been a moderate drinker and a moderate smoker. His urine was examined four or five different times, the specific gravity being 1018 to 1020; once or twice, a small quantity of sugar was found, but not enough to give evidence of much kidney trouble. The quantity of urine passed, on an average, is about five pints in twenty-four hours. His appetite is good, and he sleeps fairly well when sufficient relief from pain is obtained. His intellectual powers are not apparently impaired; there has never been any paralysis; he navigates fairly well, but gets tired very quickly.

At the present time he is being given iodide of potassium in rather large doses—one-half ounce every twenty-four hours—and is not suffering nearly as much from the intense headache. The iodide given in the above quantity for three or four days seems to produce œdema of the glottis and difficult respiration, but by omitting several doses, he is all right again.

DISCUSSION.

DR. S. G. DABNEY: I am sorry I did not hear Dr. Krim's description of the case. As far as the ocular condition is concerned, he has inflammation of both optic nerves—double optic neuritis—and a very considerably choked disc, as it is called. It is true that so much importance is not now attached to that condition of the optic nerve, as was formerly the case. It was formerly thought that when the head of the nerve protruded, it was especially indicative of increased intra-cranial pressure, probably a tumor. Although that opinion is no longer held to the same extent, it is still true that marked protrusion of the head of the nerve, in most cases, is due to some disease increasing pressure within the skull. More often than anything else, it is due to brain tumor. Of course, syphilis may produce it, independent of that. This man also had some hæmorrhages into the retina. I did not get much of his history at the time I saw him, on account of his not being able to speak English, and did not make a very thorough examination, but quite enough to be perfectly satisfied as to his condition—double optic neuritis. Inflammation of the optic nerve in brain disease, it was formerly thought, might be produced either by a descending neuritis, in which case the retina is most likely involved with the nerve, or by increased intra-cranial pressure producing well-defined protrusion of the optic disc, the so-called choked disc. Much less influence is now attributed to this latter mode of causation than formerly.

DR. W. L. RODMAN: Has there been vomiting in this case?

DR. J. M. KRIM: No vomiting at all, not even nausea.

DR. T. C. EVANS: I examined this patient about four weeks ago. At that time he was blind; at least, his vision was reduced to a perception of light. It was rather a difficult case to examine with the ophthalmoscope—on account of the intense photophobia, the eye could hardly be kept open long enough to make an ophthalmoscopical examination. At that time he had a decided optic neuritis in both eyes, with a more or less choked disc and with a considerable protrusion of the head of the nerve, as Dr. Dabney has said; also a few hæmorrhagic spots in the retina. It was impossible for me to get any history of the case, as he understands very little English, and I do not speak German at all; consequently, I could only get what I could see. From the objective symptoms it was plainly a case of double optic neuritis. While he is not paralyzed in anyway, still I noticed then, the same as now, that after sitting quietly for a short time, there is a peculiar sighing respiration; this, I think, is very significant.

In regard to the case of optic neuritis, I believe the idea has been generally abandoned now that it is due to the intra-cranial pressure. It was formerly thought that this was the cause, from the simple fact that cases have occurred with brain tumor, and the tumor itself was evidence that it was due to intra-cranial pressure. Now it is recognized, I think, by most authorities, as being due to an inter-current affection, but just what produces the inflammation, I do not know.

DR. J. W. IRWIN: What has been the rate of the pulse and temperature.

DR. J. M. KRIM: The temperature is practically normal, pulse rapid.

DR. J. W. IRWIN: This is certainly a very interesting case, and it is in line with one presented by Dr. Wm. Cheatham, recently, before the society, in which the doctor had diagnosticated brain tumor; and at the same time, the patient manifested very marked symptoms of locomotor ataxia. An investigation was made before the society, and a pronounced absence of the patella tendon reflex was discovered. Subsequen examination developed the fact that there was no absence of this reflex. The case before us presents much more pronounced evidences of brain tumor than the one shown by Dr. Cheatham. The expression of this man is striking; so also is his walk. In such cases, we are apt to have paroxysmal pain; again, absence of pain. This feature seems to be very marked in the case before us. It is hard to account for the sudden loss of sight in this case, coming on while using a sewing machine; possibly some effusion took place at that time, producing localized pressure, but this view is surmise only. From the history of the case, I am inclined to the belief that there is a small tumor near the base of the brain, pressing on the optic tract at some point far back, not being sufficiently large to produce disturbance of any other part. The trouble may possibly be caused by serious pressure due to chronic meningitis, which would, in a measure, account for the pain. The absence of fever does not prove that meningitis may not be present, but this and the symptoms present go to show that meningitis is not extensive, and, if a factor, it may be limited to the immediate surroundings of the neoplasm.

